# Supplementary material for: Minding the knowledge-action gap: Results from a mixed-methods study of antimicrobial use among dairy farmers in central Uganda
Source: PLoS One. 2026 Jan 9;21(1):e0339969. doi: 10.1371/journal.pone.0339969 (PMC12788652; doi:10.1371/journal.pone.0339969)
Supplement: S1 Annex A — (DOCX) [file pone.0339969.s001.docx]

**ANNEX A**

**Descriptive statistics, LASSO results and model diagnostics**

**Table S1. Descriptive statistics of binary and categorical variables entered into the LASSO regressions.** AHP stands for “Animal Health Professional”, including both government and private veterinarians.

| **Model name** | **Variable Definition and categories** | **Freq.** | **Percent** |
| --- | --- | --- | --- |
| **Age** | **Age** |  |  |
|  | 18-24 | 36 | 8.63 |
|  | 25-30 | 51 | 12.23 |
|  | 31-40 | 97 | 23.26 |
|  | 41-50 | 105 | 25.18 |
|  | 51-60 | 68 | 16.31 |
|  | >60 | 60 | 14.39 |
| **Gender** | **Gender** |  |  |
|  | Male | 252 | 60.43 |
|  | Female | 165 | 39.57 |
| **Education** | **Highest Education** |  |  |
|  | Primary | 197 | 47.24 |
|  | Secondary | 167 | 40.05 |
|  | Tertiary | 53 | 12.71 |
| **Additional training** | **Training related to animal production/health** |  |  |
|  | No | 302 | 72.42 |
|  | Yes | 115 | 27.58 |
| **Dairy>50 income** | **Dairy Contributes over 50% of income** |  |  |
|  | No | 253 | 60.67 |
|  | Yes | 164 | 39.33 |
| **Dairy_keeping** | **Years keeping dairy** |  |  |
|  | Under 2 years | 16 | 3.84 |
|  | 2-5 years | 71 | 17.03 |
|  | 6-10 years | 121 | 29.02 |
|  | 11-20 years | 118 | 28.30 |
|  | 21 to 30 years | 54 | 12.95 |
|  | Over thirty years | 37 | 8.87 |
| **Grazing** | **Herd Free Range or Zero Grazing(1=Free-Range)** |  |  |
|  | No | 286 | 68.59 |
|  | Yes | 131 | 31.41 |
| **Professional AH Source** | **Seek out advice from animal health professionals** |  |  |
|  | No | 51 | 12.23 |
|  | Yes | 366 | 87.77 |
| **Informal AH source** | **Whether farmer reported to get animal health advice from informal source** |  |  |
|  | No | 232 | 55.64 |
|  | Yes | 185 | 44.36 |
| **Correct AM Source** |  |  |  |
|  | No | 120 | 28.78 |
|  | Yes | 297 | 71.22 |
| **Define AMR** | **Can correctly define AMR** |  |  |
|  | No | 314 | 75.30 |
|  | Yes | 103 | 24.70 |
| **Withdrawal Information** | **Whether respondent reported being told by an animal health professional about the importance of observing antibiotic withdrawal periods in milk consumption and sales** |  |  |
|  | No | 124 | 29.74 |
|  | Yes | 293 | 70.26 |
| **Misuse Information** | **Have been told by an AHP about the issue of drug misuse** |  |  |
|  | No | 131 | 31.41 |
|  | Yes | 286 | 68.59 |
| **Bigger_Faster** | **“Injectables” can help cattle grow bigger/faster** |  |  |
|  | disagree | 286 | 68.59 |
|  | agree | 131 | 31.41 |
| **AM Overuse** | **If injectables are given too often, they might stop working** |  |  |
|  | disagree | 136 | 32.61 |
|  | agree | 281 | 67.39 |
| **Stop Treat Agree** | **You can stop giving cow full course if symptoms improve** |  |  |
|  | disagree | 228 | 54.68 |
|  | agree | 189 | 45.32 |
| **Prevent agree** | **Giving healthy cattle injectables will prevent future sickness** |  |  |
|  | disagree | 286 | 68.59 |
|  | agree | 131 | 31.41 |
| **Withdrawal agree** | **After using veterinary drug on an animal, you should wait to use milk** |  |  |
|  | No | 110 | 26.38 |
|  | Yes | 307 | 73.62 |
| **Vet Accessibility** | **Animal health professionals are easily accessible** |  |  |
|  | disagree | 48 | 11.57 |
|  | agree | 367 | 88.43 |
| **No farm records** | **Farmer does not keep any farm records** |  |  |
|  | No | 200 | 47.96 |
|  | Yes | 217 | 52.04 |
| **Satisfaction Priv. Vets** | **Farmer reports to be satisfied with animal healthcare services provided by private veterinarians** |  |  |
|  | No | 127 | 30.4 |
|  | Yes | 290 | 69,54 |
| **Satisfaction Gov. Vets** | **Farmer reports to be satisfied with animal healthcare services provided by private veterinarians** |  |  |
|  | No | 305 | 73.14 |
|  | Yes | 112 | 26.86 |
| **Vet at interview** | **Veterinarian was present at farm during interview** |  |  |
|  | No | 222 | 53.37 |
|  | Yes | 194 | 46,63 |

**Table S2. Descriptive statistics of continuous variables entered into the LASSO regressions**

| **Model Name** | **Variable Definition** | **Mean** | **Std. Dev.** | **Min- Max** |
| --- | --- | --- | --- | --- |
| Herd_Size | Dairy Herd Size | 4.254 | 6.471 | 1-100 |
| Diseases | Number of diseases farmer reported as common. See Table SX for common diseases | 2.05 | 1.369 | 0-6 |
| Num incorrect AH source | Number of non-AHP consulted for animal health advice | .619 | .797 | 0-4 |
| Num. Vet_challenges | Number of challenges to accessing vets  (cost, distance, poor network, not aware of vet, unavailable) | 1.125 | .882 | 0-3 |

**Table S2. LASSO Knots Table: Sick animals are treated by animal health professionals (public or private)** * Indicates lambda selected by cross-validation

| **ID** | **lambda** | **No of nonzero coef.** | **CV mean prediction error** | | **Variables (A)dded, (R)emoved or left (U)nchanged** |
| --- | --- | --- | --- | --- | --- |
| 1 | 0.081188 | 1 | 1.230698 | A | vet_present |
| 2 | 0.073976 | 3 | 1.228541 | A | no.informal_ah |
|  |  |  |  |  | num_vet_challenges |
| 5 | 0.05596 | 4 | 1.211027 | A | age |
| *8 | 0.042332 | 6 | 1.19882 | A | disease_burden Vet_Accessible |
| 12 | 0.029178 | 6 | 1.194069 | U |  |
| 13 | 0.026585 | 8 | 1.19481 | A | livestockkeep correct_ah_info |
| 14 | 0.024224 | 10 | 1.195574 | A | farmrecords1 agreeprevention |
| 15 | 0.022072 | 12 | 1.196329 | A | agreegrowth agreesymptoms |
| 16 | 0.020111 | 12 | 1.196469 | U |  |

**Table S3. LASSO Knots Table: Call Veterinarian Prior to Any Treatment of Sick Animals**. * Indicates lambda selected by cross-validation

| **ID** | **lambda** | **No of nonzero coef.** | **CV mean prediction error** |  | **Variables (A)dded, (R)emoved or left (U)nchanged** |
| --- | --- | --- | --- | --- | --- |
| 1 | 0.088808 | 1 | 0.640199 | A | vet_present |
| 2 | 0.080919 | 2 | 0.635367 | A | correct_ah_info |
| 12 | 0.031916 | 3 | 0.583179 | A | agreesymptoms |
| 14 | 0.026497 | 4 | 0.582299 | A | Vet_Accessible |
| 17 | 0.020044 | 6 | 0.58498 | A | agreeprevention |
|  |  |  |  |  | num_vet_challenges |
| *18 | 0.018264 | 6 | 0.586053 | U |  |

**Table S4. LASSO Knots Table: Milk is properly disposed during withdrawal period**. * Indicates lambda selected by cross-validation

| **ID** | **lambda** | **No of nonzero coef.** | **CV mean prediction error** |  | **Variables (A)dded, (R)emoved or left (U)nchanged** |
| --- | --- | --- | --- | --- | --- |
| 1 | 0.04532 | 1 | 0.735895 | A | vet_present |
| 2 | 0.041294 | 3 | 0.734457 | A | disease_burden |
|  |  |  |  |  | withdrawalinfo |
| *10 | 0.019618 | 4 | 0.711903 | A | no_informal_ah |
| 11 | 0.017875 | 5 | 0.71234 | A | agreewithdrawal |
| 12 | 0.016287 | 6 | 0.713631 | A | num_vet_challenges |
| 14 | 0.013522 | 7 | 0.718322 | A | livestock_50 |

**Table S5. LASSO Knots Table: Correct Dosage always given (not smaller or larger dose than recommended).** * Indicates lambda selected by cross-validation. ♦ Indicates where visual inspection of “elbow points” were used to select more parsimonious set of predictors.

| **ID** | **lambda** | **No of nonzero coef.** | **CV mean prediction error** | | **Variables (A)dded, (R)emoved or left (U)nchanged** |
| --- | --- | --- | --- | --- | --- |
| 1 | 0.100163 | 1 | 1.164916 | A | vet_present |
| 2 | 0.091265 | 3 | 1.15616 | A | agreesymptoms num_vet_challenges |
| 5 | 0.069039 | 4 | 1.12273 | A | agreeprevention |
| 6 | 0.062906 | 6 | 1.113816 | A | farmrecords1 disease_burden |
| 7 | 0.057317 | 7 | 1.104142 | A | correct_ah_info |
| 9 | 0.047586 | 8 | 1.083955 | A | grazing |
| 10 | 0.043358 | 9 | 1.074826 | A | correct_am_source |
| 11 | 0.039507 | 10 | 1.066846 | A | knowamr |
| ♦12 | 0.035997 | 11 | 1.059605 | A | age |
| 15 | 0.02723 | 12 | 1.044248 | A | gender |
| 16 | 0.024811 | 13 | 1.041165 | A | agreeresistance |
| 18 | 0.020599 | 15 | 1.036849 | A | agreegrowth livestockkeep |
| 20 | 0.017101 | 16 | 1.032285 | A | livestock_50 |
| 22 | 0.014198 | 18 | 1.028998 | A | edu_additional num_incorrect_ah_info |
| *27 | 0.008917 | 18 | 1.026215 | U |  |
| 30 | 0.006745 | 19 | 1.026634 | A | Herd_Size |
| 31 | 0.006146 | 20 | 1.026868 | A | Vet_Accessible |
| 33 | 0.005103 | 21 | 1.027542 | A | education |
| 35 | 0.004236 | 21 | 1.028384 | U |  |

**Table S6. LASSO Knots Table: Whether an animal health professional (public or private) is usually sought for general animal health information.** * Indicates lambda selected by cross-validation. ♦ Indicates where visual inspection of “elbow points” were used to select more parsimonious set of predictors.

| **ID** | **lambda** | **No of nonzero coef.** | **CV mean prediction error** |  | **Variables (A)dded, (R)emoved or left (U)nchanged** |
| --- | --- | --- | --- | --- | --- |
| 1 | 0.089335 | 1 | 0.75201 | A | vet_present |
| 2 | 0.081399 | 2 | 0.745184 | A | num_incorrect_ah_info |
| 6 | 0.056105 | 3 | 0.709493 | A | 3.sub_county_cat_nums |
| 11 | 0.035236 | 6 | 0.687148 | A | amrinfo 12.sub_county_cat_nums |
|  |  |  |  |  | satisfication_priv_vets |
| 13 | 0.029253 | 9 | 0.681747 | A | grazing livestock_50 disease_burden |
| ♦15 | 0.024287 | 10 | 0.673568 | A | knowamr |
| 17 | 0.020163 | 12 | 0.664487 | A | education livestockkeep |
| 19 | 0.01674 | 14 | 0.657175 | A | Vet_Accessible |
|  |  |  |  |  | 8.sub_county_cat_nums |
| *23 | 0.011538 | 14 | 0.651726 | U |  |
| 26 | 0.008728 | 17 | 0.656999 | A | edu_additional |
|  |  |  |  |  | 1.sub_county_cat_nums |
|  |  |  |  |  | 7.sub_county_cat_nums |
| 27 | 0.007953 | 17 | 0.660752 | U |  |

**Table S7. LASSO Knots Table: Whether an informal source of general animal health information is usually sought, including friends, family, other farmers, internet, etc.** * Indicates lambda selected by cross-validation. ♦ Indicates where visual inspection of “elbow points” were used to select more parsimonious set of predictors.

| **ID** | **lambda** | **No of nonzero coef.** | **CV mean prediction error** | | **Variables (A)dded, (R)emoved or left (U)nchanged** |
| --- | --- | --- | --- | --- | --- |
| 1 | 0.203431 | 1 | 1.329661 | A | vet_present |
| 2 | 0.185359 | 2 | 1.30293 | A | correct_ah_info |
| 10 | 0.08806 | 3 | 1.169474 | A | 4.sub_county_cat_nums |
| 11 | 0.080237 | 4 | 1.157709 | A | disease_burden |
| 14 | 0.060697 | 5 | 1.122516 | A | 12.sub_county_cat_nums |
| 16 | 0.050391 | 6 | 1.101821 | A | edu_additional |
| 19 | 0.038119 | 7 | 1.075213 | A | amrinfo |
| 20 | 0.034733 | 8 | 1.067796 | A | 13.sub_county_cat_nums |
| ^♦^22 | 0.028836 | 9 | 1.055203 | A | 1.sub_county_cat_nums |
| 25 | 0.021813 | 13 | 1.047912 | A | age livestock_50 |
|  |  |  |  |  | num_vet_challenges |
|  |  |  |  |  | 9.sub_county_cat_nums |
| 26 | 0.019875 | 14 | 1.047371 | A | livestockkeep |
| *27 | 0.01811 | 14 | 1.047363 | U |  |
| 29 | 0.015035 | 16 | 1.04906 | A | gender satisfication_priv_vets |
| 31 | 0.012482 | 16 | 1.051496 | U |  |

**Table S8. LASSO Knots Table: Whether challenges accessing vets were experienced**. * Indicates lambda selected by cross-validation. ♦ Indicates where visual inspection of “elbow points” were used to select more parsimonious set of predictors.

| **ID** | **lambda** | **No of nonzero coef.** | **CV mean prediction error** | | **Variables (A)dded, (R)emoved or left (U)nchanged** |
| --- | --- | --- | --- | --- | --- |
| 1 | 0.095426 | 1 | 1.016459 | A | vet_present |
| 2 | 0.086949 | 2 | 1.012351 | A | 4.sub_county_cat_nums |
| 5 | 0.065773 | 3 | 0.996152 | A | disease_burden |
| 7 | 0.054606 | 4 | 0.983357 | A | livestock_50 |
| 8 | 0.049755 | 6 | 0.974472 | A | age grazing |
| 9 | 0.045335 | 7 | 0.964158 | A | 1.incorrect_ah_cats |
| 10 | 0.041308 | 8 | 0.954464 | A | 7.sub_county_cat_nums |
| 11 | 0.037638 | 10 | 0.944695 | A | 10.sub_county_cat_nums |
|  |  |  |  |  | 13.sub_county_cat_nums |
| ♦12 | 0.034294 | 11 | 0.935371 | A | 2.sub_county_cat_nums |
| 16 | 0.023638 | 12 | 0.907114 | A | satisfication_priv_vets |
| 17 | 0.021538 | 15 | 0.902983 | A | Herd_Size 8.sub_county_cat_nums |
|  |  |  |  |  | 9.sub_county_cat_nums |
| 19 | 0.017881 | 16 | 0.896747 | A | gender |
| *23 | 0.012325 | 17 | 0.889888 | A | 1.sub_county_cat_nums |
| 26 | 0.009323 | 18 | 0.892812 | A | 3.sub_county_cat_nums |
| 27 | 0.008495 | 18 | 0.894742 | U |  |

**Table S9. Full Model for whether recommended dosage was reportedly always followed.** Models include all variables selected in LASSO regressions using 10-fold cross-validation.

| Correlates | Observe Correct Dosage |
| --- | --- |
| Vet at Interview | 0.308*** |
|  | (0.175 - 0.540) |
| Age | 1.265* |
|  | (1.047 - 1.528) |
| Gender | 1.487 |
|  | (0.860 - 2.573) |
| Grazing | 0.650 |
|  | (0.375 - 1.127) |
| Dairy Keep | 0.861 |
|  | (0.691 - 1.075) |
| Additional training | 0.739 |
|  | (0.404 - 1.351) |
| Diseases | 0.794* |
|  | (0.642 - 0.983) |
| Correct AH source | 2.231* |
|  | (1.028 - 4.842) |
| Correct AM Source | 0.703 |
|  | (0.356 - 1.391) |
| Define AMR | 0.635 |
|  | (0.352 - 1.146) |
| Withdrawal Agree | 0.761 |
|  | (0.434 - 1.334) |
| Bigger/Faster Agree | 1.538 |
|  | (0.830 - 2.849) |
| Prevent Agree | 0.484* |
|  | (0.267 - 0.876) |
| Stop Treat Agree | 0.428** |
|  | (0.240 - 0.761) |
| Num Vet Challenges | 0.601** |
|  | (0.434 - 0.833) |
| Dairy>50 income | 1.398 |
|  | (0.800 - 2.444) |
| No Farm Records | 0.467** |
|  | (0.269 - 0.813) |
| No non-AHP sources | 0.969 |
|  | (0.662 - 1.419) |
| Constant | 23.411*** |
|  | (5.237 - 104.660) |
|  |  |
| Observations | 416 |

Robust 95% CI in parentheses

*** p<0.001, ** p<0.01, * p<0.05

**Table S10. Full Models for animal health seeking practices**. Models include all variables selected in LASSO regression using 10-fold cross-validation.

| VARIABLES | Professional AH  Sources | Informal AH  Sources | Vet Challenges  Experienced |
| --- | --- | --- | --- |
|  |  |  |  |
| Age |  | 0.928 | 0.795* |
|  |  | (0.801 - 1.076) | (0.658 - 0.959) |
| Additional training |  | 1.909* |  |
|  |  | (1.151 - 3.165) |  |
| Num. Vet Challenges |  | 1.285 |  |
|  |  | (0.983 - 1.681) |  |
| Vet at Interview | 0.645 | 0.315*** | 2.057* |
|  | (0.260 - 1.599) | (0.185 - 0.537) | (1.094 - 3.868) |
| Informal AH Source | 0.234*** |  | 2.374** |
|  | (0.135 - 0.408) |  | (1.234 - 4.568) |
| Professional AH Source |  | 0.275* |  |
|  |  | (0.102 - 0.743) |  |
| Education | 1.401 |  |  |
|  | (0.744 - 2.637) |  |  |
| Gender |  |  | 1.185 |
|  |  |  | (0.676 - 2.076) |
| Herd Size |  |  | 0.964 |
|  |  |  | (0.922 - 1.007) |
| Dairy>50 income | 0.762 | 1.681* | 2.649** |
|  | (0.350 - 1.659) | (1.044 - 2.706) | (1.468 - 4.780) |
| Dairy Keep | 0.757 | 1.040 |  |
|  | (0.548 - 1.045) | (0.868 - 1.246) |  |
| Grazing | 0.440* |  | 1.926 |
|  | (0.206 - 0.941) |  | (0.952 - 3.898) |
| Diseases | 1.566* | 1.218* | 1.703*** |
|  | (1.027 - 2.385) | (1.010 - 1.470) | (1.308 - 2.218) |
| AHP Access | 1.654 |  |  |
|  | (0.467 - 5.862) |  |  |
| Define AMR | 0.596 |  |  |
|  | (0.284 - 1.248) |  |  |
| Misuse Information | 0.307* | 0.640 |  |
|  | (0.095 - 0.996) | (0.368 - 1.110) |  |
| Satisfaction Priv. Vets | 1.529 |  | 0.578 |
|  | (0.880 - 2.656) |  | (0.328 - 1.020) |
| Sub_county_1 |  | 1.316 | 2.239 |
|  |  | (0.528 - 3.278) | (0.463 - 10.823) |
| Sub_county_2 |  | 0.295** | 0.569 |
|  |  | (0.139 - 0.624) | (0.256 - 1.267) |
| Sub_county_3 | 0.206** | 1.708 | 0.316 |
|  | (0.080 - 0.533) | (0.830 - 3.513) | (0.075 - 1.326) |
| Sub_county_4 |  | 0.387 | 4.867 |
|  |  | (0.093 - 1.619) | (0.976 - 24.260) |
| Sub_county_5 |  |  | 0.186 |
|  |  |  | (0.016 - 2.188) |
| Sub_county_8 | 3.163 |  | 0.692 |
|  | (0.507 - 19.746) |  | (0.330 - 1.447) |
| Sub_county_9 | 0.343 | 1.708 | 0.316 |
|  | (0.100 - 1.175) | (0.830 - 3.513) | (0.075 - 1.326) |
| Sub_county_10 |  |  | 1.967 |
|  |  |  | (0.647 - 5.980) |
| Sub_county_13 |  | 0.254** | 7.146** |
|  |  | (0.100 - 0.645) | (1.779 - 28.701) |
| Constant | 52.204** | 2.982 | 2.319 |
|  | (3.986 - 683.664) | (0.800 - 11.118) | (0.513 - 10.489) |
|  |  |  |  |
| Observations | 402 | 416 | 402 |

**Table S11. Area under curve, percentage of correctly classified, and Hosmer-Lemeshow values with associated significance tests**.

| **Model** | **AUC** | **Correctly Classified** | **Hosmer-Lemeshow** | **HL: Prob> chi2** |
| --- | --- | --- | --- | --- |
| Correct Treater | 0.7284 | 69.81% | 8.83 | 0.3565 |
| Call Vet First | 0.7975 | 89.15% | 6.65 | 0.5743 |
| Withdrawal Correct | 0.7004 | 88.22% | 5.46 | 0.7072 |
| Correct Dosage | 0.8197 | 75.72% | 7.34 | 0.5008 |
| Correct AH Info | 0.8573 | 89.29% | 9.36 | 0.3125 |
| Incorrect AH Info | 0.7445 | 69.71% | 7.96 | 0.4376 |
| Vet Challenges | 0.7992 | 79.90% | 4.94 | 0.7635 |

**Table S12.** **Variance Inflation Factors associated with the antimicrobial use and related practices models.**

| **Model** | **Variable** | **VIF** | **Tolerance** |
| --- | --- | --- | --- |
| **Correct Treater** | vet_present | 1.09 | 0.917352 |
|  | age | 1.03 | 0.974736 |
|  | diseases | 1.12 | 0.893396 |
|  | informal | 1.1 | 0.906809 |
|  | num_vet_ch~s | 1.17 | 0.852481 |
|  | Vet_Access~e | 1.07 | 0.932673 |
| **First Treatment Step** |  |  |  |
|  | agreesympt~s | 1.22 | 0.82041 |
|  | agreepreve~n | 1.19 | 0.841568 |
|  | num_vet_ch~s | 1.11 | 0.90088 |
|  | Vet_Access~e | 1.1 | 0.9115 |
|  | vet_present | 1.06 | 0.945104 |
|  | correct_ah~o | 1.03 | 0.973506 |
| **Observe Withdrawal** |  |  |  |
|  | vet_present | 1.09 | 0.913825 |
|  | disease_bu~n | 1.12 | 0.890654 |
|  | withdrawal~o | 1.03 | 0.96625 |
|  | informal | 1.17 | 0.854215 |
| **Observe Correct Dosage** |  |  |  |
|  | agreesympt~s | 1.3 | 0.771293 |
|  | correct_am~e | 1.29 | 0.777756 |
|  | num_vet_ch~s | 1.26 | 0.791714 |
|  | disease_bu~n | 1.24 | 0.804076 |
|  | agreepreve~n | 1.21 | 0.823367 |
|  | age | 1.18 | 0.849861 |
|  | grazing | 1.16 | 0.863536 |
|  | farmrecords1 | 1.16 | 0.864915 |
|  | livestockk~p | 1.14 | 0.875585 |
|  | vet_present | 1.14 | 0.878332 |
|  | agreeresis~e | 1.12 | 0.893983 |
|  | knowamr | 1.12 | 0.895672 |
|  | gender | 1.1 | 0.906618 |
|  | formal | 1.09 | 0.920618 |

**Table S13. Variance Inflation Factors associated with animal health seeking models.**

| Model | Variabl | VIF | Tolerance |
| --- | --- | --- | --- |
| Correct AH Info | vet_present | 1.26 | 0.795214 |
|  | informal | 1.2 | 0.834771 |
|  | livestock_50 | 1.06 | 0.946443 |
|  | grazing | 1.11 | 0.90361 |
|  | disease_bu~n | 1.19 | 0.838701 |
|  | amrinfo | 1.25 | 0.80019 |
|  | sati~iv_vets | 1.04 | 0.960821 |
|  | sub_county~s |  |  |
|  | 3 | 1.14 | 0.880738 |
|  | 12 | 1.12 | 0.895063 |
| Incorrect Treater |  |  |  |
|  | vet_present | 1.28 | 0.782833 |
|  | formal | 1.14 | 0.87938 |
|  | edu_additi~l | 1.06 | 0.947802 |
|  | disease_bu~n | 1.14 | 0.873991 |
|  | amrinfo | 1.32 | 0.7585 |
|  | sub_county~s |  |  |
|  | 1 | 1.08 | 0.926721 |
|  | 4 | 1.34 | 0.746632 |
|  | 12 | 1.21 | 0.824953 |
|  | 13 | 1.23 | 0.812518 |
| Vet Challenges |  |  |  |
|  | age | 1.01 | 0.986137 |
|  | livestock_50 | 1.05 | 0.948453 |
|  | grazing | 1.05 | 0.948726 |
|  | disease_bu~n | 1.1 | 0.908838 |
|  | sub_county~s |  |  |
|  | 4 | 1.11 | 0.902489 |
|  | 10 | 1.02 | 0.985148 |
|  | 13 | 1.09 | 0.917394 |
|  | satisfaction_vets | 1.04 | 0.959217 |
|  | informal | 1.06 | 0.941328 |

**Figure S1. Leverage by predicted probabilities for Correct Treater model.**

**Figure S2. Pearson residuals by predicted probabilities for Correct Treater model.**

**Figure S3. Deviance residuals by predicted probabilities for Correct Treater model.**

**Figure S4. Leverage by predicted probabilities for First Step Treatment model.**

**Figure S5. Pearson residuals by predicted probabilities for First Step Treatment model.**

**Figure S6. Deviance residuals by predicted probabilities for First Step Treatment model.**

**Figure S7. Leverage by predicted probabilities for Observe Withdrawal model**.

**Figure S8. Pearson residuals by predicted probability for Observe Withdrawal model.**

**Figure S9. Deviance residuals by predicted probability for Observe Withdrawal model.**

**Figure S10. Leverage by predicted probabilities for Observe Correct Dosage model.**

**Figure S11. Pearson residuals by predicted probability for Observe Correct Dosage model.**

**Figure S12. Deviance residuals by predicted probability for Observe Correct Dosage model.**

**Figure S13. Leverage by predicted probabilities for Professional AH Sources model.**

**Figure S14. Pearson residuals by predicted probability for Professional AH Sources model.**

**Figure S15. Deviance residuals by predicted probability for Professional AH Sources model**

**Figure S16. Leverage by predicted probabilities for Informal AH Sources model.**

**Figure S17. Pearson residuals by predicted probability for Informal AH Sources model.**

**Figure S18. Deviance residuals by predicted probability for Informal AH Sources model**

**Figure S19. Leverage by predicted probabilities for Veterinary Challenges model.**

**Figure S20. Pearson residuals by predicted probability for Veterinary Challenges model**

**Figure S21. Deviance residuals by predicted probability for Veterinary Challenges model**
