# Supplementary material for: Minding the knowledge-action gap: Results from a mixed-methods study of antimicrobial use among dairy farmers in central Uganda
Source: PLoS One. 2026 Jan 9;21(1):e0339969. doi: 10.1371/journal.pone.0339969 (PMC12788652; doi:10.1371/journal.pone.0339969)
Supplement: S2 Annex B — (DOCX) [file pone.0339969.s002.docx]

**ANNEX B**

**Power analysis for sample size estimation and**

**F tests** - Linear multiple regression: Fixed model, R² deviation from zero

**Analysis:** A priori: Compute required sample size

**Input:** Effect size f² = 0.04

α err prob = 0.05

Power (1-β err prob) = 0.8

Number of predictors = 10

**Output:** Noncentrality parameter λ = 16.6400000

Critical F = 1.8540972

Numerator df = 10

Denominator df = 405

Total sample size = 416

Actual power = 0.8008883
