# Supplementary material for: Minding the knowledge-action gap: Results from a mixed-methods study of antimicrobial use among dairy farmers in central Uganda
Source: PLoS One. 2026 Jan 9;21(1):e0339969. doi: 10.1371/journal.pone.0339969 (PMC12788652; doi:10.1371/journal.pone.0339969)
Supplement: S3 Annex C — (DOCX) [file pone.0339969.s003.docx]

**ANNEX C**

**Qualitative Codebook**

| Code | Definition |
| --- | --- |
| vet | References to veterinarians/animal health workers and their role, actions, guidance, or availability. |
| self-treatment | Farmers, family members, herdsmen, or neighbors diagnose or treat animals without a vet. |
| AMR | Perceived drug/acaricide failure or need to increase dosage/change products because the drugs no longer work. |
| withdrawal | Statements about not consuming or selling milk/meat after treatment, or ignoring withdrawal advice. |
| selling | Selling or slaughtering animals because they are sick or likely to die. |
| cost | Cost of vets, drugs, or treatment causes difficulty, delay, or inability to follow recommendations. |
| prescription | The vet instructs which drug/dose to use but does not administer directly. |
| biosecurity | Practices aimed at preventing or reducing disease spread (hygiene, PPE, milking practices). |
| information | Sources of animal-health knowledge (e.g., radio, neighbors, vets, workshops, TV, experience). |
| conflicting information | Advice from different sources is contradictory or confusing. |
| lack of follow-up | Vets/researchers do not return to check progress or provide continued support. |
| mandate | Official or expected roles of vets (e.g., government vs private) and boundaries of responsibility. |
| supernatural | Reliance on God/divine protection instead of management practices for animal health. |
| treatment followed | Speaker emphasizes completing or correctly following vet instructions. |
| context | Advice or recommended practice does not fit local environmental, economic, or cultural conditions. |
