# Supplementary material for: Minding the knowledge-action gap: Results from a mixed-methods study of antimicrobial use among dairy farmers in central Uganda
Source: PLoS One. 2026 Jan 9;21(1):e0339969. doi: 10.1371/journal.pone.0339969 (PMC12788652; doi:10.1371/journal.pone.0339969)
Supplement: S4 Annex D — (DOCX) [file pone.0339969.s004.docx]

**ANNEX C**

**KAP Instrument**

# Study Introduction

Hello, my name is and I'm working with the College of Veterinary Medicine at Makerere University in collaboration with the Food and Agricultural Organization on a project looking at dairy cattle health in Greater Kampala

We are interested in knowing how you manage your farm and we want to know this because we want to develop a program to help you improve the health of your animals

This survey will take about 45 mins to 1 hr. of your time

You do not have to agree to be interviewed and you can stop at any time choose and there will be no negative consequences for you

Anything you say during the discussion today will remain completely conﬁdential: your name will not be used in any materials produced from this study nor any other information that could be used to identify me

Data obtained will be used in writing reports, publications and presentations in conferences and workshops, but any personal data will not be displayed.

Do you have any questions before we proceed?

## I agree to take part of this study


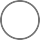
 Yes
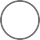
 No

## Input ID number from consent form

## Please check District


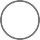
 Kampala
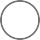
 Mukono
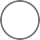
 Wakiso

**Please write Sub-county**

**Please write Village name**

**Please write Parish**

1. **Respondent position in the household**


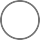
 household head


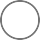
 spouse of household head
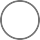
 son


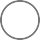
 daughter
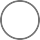
 Employee
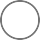
 Relative
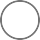
 Other

## Specify other.

1. **What is your age?**


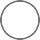
 18-24


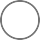
 25-30


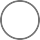
 31-40


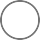
 41-50


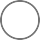
 51-60


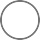
 >60

## Please select gender


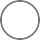
 Male
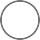
 Female

## What level of education does the respondent have?


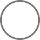
 primary
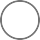
 secondary
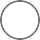
 Tertiary


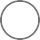
 No formal education but adult education

## Can anyone in the household read and write?


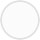
 Yes
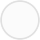
 No

## What is the <b>MAIN SOURCE</b> of income for the household?


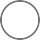
 Dairy farming
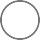
 Other livestock
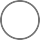
 Crop products


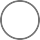
 Salaried Employment
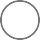
 Self-Employed-Oﬀ Farm
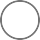
 Other

## Specify other.

1. **How much does livestock contribute to household income?**


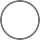

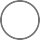
 To half or more of the household's income
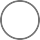
 To less than half of the household's income
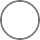
 Does not contribute to the household income

I don't know

## How long has the household been keeping livestock?


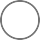
 Under 2 years


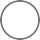
 2-5 years


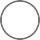
 6-10 years


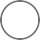
 11-20 years


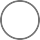
 21 to 30 years
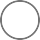
 Over thirty years

## How do you USUALLY keep your dairy cattle?


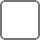
 Free ranging
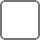
 Paddocking


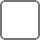
 Zero grazing/feed lot
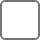
 Tethering


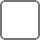
 Other

**Specify other.**

1. **How many dairy cattle do you own?**
2. **What breeds do you keep?**


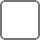
 crossed


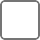
 pure breed exotic
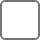
 pure breed local

## How many dairy cattle have you acquired in the last year? (If none, put the number 0)

*Calves included. They could also buy and cattle died so ask EVEN IF they have no cattle currently.*

## What other animals do you keep?


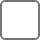
 Beef cattle
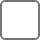
 Bees


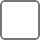
 Chicken


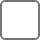
 Companion animals
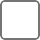
 Goats


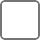
 Pigs


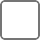
 Other poultry
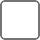
 Sheep


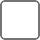
 None
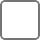
 Other

## Specify other.

1. **Do these animals interact with the dairy cattle? (Habitation/grazing)**


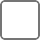
 Yes, they live close together
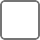
 Yes, they graze together
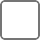
 No

## How often are they grazing together?


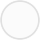
 Daily


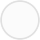
 At least once a week
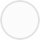
 At least once a month

## Where did you get/acquire the dairy animals you received the last year?

*Select all that apply*


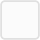
 Livestock Market
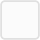
 Family


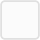
 Neighbors/Friends
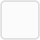
 Livestock breeders
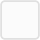
 Other farmers


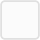
 Government development programs
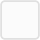
 I don't get any dairy cattle


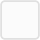
 Other

## Specify other.

1. **What do you USUALLY do when introducing a new animal to your herd?**

*Select all that apply*


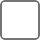
 Animal identiﬁcation (Tagging, branding, naming, tattooing)


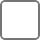
 Called a veterinarian / animal health oﬃcer for a health check / advice
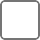
 Checked whether it was vaccinated / got it vaccinated


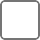
 Dewormed / sprayed / provided it with vitamins


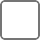
 Got information on the animal health status before purchase
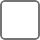
 Immediately mixed it with the other animals


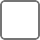
 Isolate the new animal and observe it for disease signs (e.g. protection)
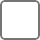
 Opened a health book


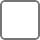
 Treat and mix it with the rest of the herd
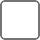
 Other

## Specify other.

1. **What do you USUALLY do with waste from your dairy farm?**

*Select all that apply*


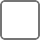
 Dry and burn
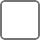
 Fertilize ﬁelds
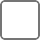
 For biogas


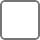
 Preparation of compost heap (intentional)
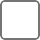
 Sell it/Give it to Neighbors


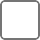
 Throw it away (unintentional compost)

## What are the <b>MAIN SOURCES</b> of water your livestock use throughout the year?

*Select all that apply*


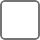
 Borehole/protected springs
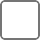
 Open well


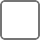
 Protected well
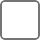
 Rain water
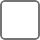
 Tap water


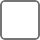
 Vendors


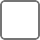
 Water pans/dams
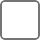
 Other

## Specify other.

1. **Do livestock and wildlife USUALLY get water from the same source?**


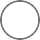
 Yes
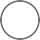
 No

## Do livestock and humans USUALLY drink water from the same source?

Yes No

# Now, I would like to ask you a few questions about the health of your dairy cattle

## What are the MOST COMMON diseases your dairy cattle get?

*Select all that apply*

My animals never get sick Brucellosis (Bulusela)

Fever (omusuja)- Anaplasmosis, Babesiosis and other causes of fever East Coast Fever (Makebe)

Foot and Mouth Disease (Kalusu) Lumpy Skin Diseases (kifuluto) Mastitis (Ebanyi)

Pneumonia (senyiga) Anthrax

Rabies

Eye infections (blindness) Foot rot

Worm Infestation (enjoka) Mange, ring worms (olukuku) Milk fever

Tetanus

I don't know Other

## Specify other.

1. **Has there been any mortality of dairy cattle in your farm within the last one year?**

Yes No

## When a dairy cattle has died, what do you do with it?

*Select all that apply*

Consume within household and/or neighbour/freinds Sell it for slaughter (Butchery/Traders)

Give it to dogs Bury it

Burn it

Report to vet

Throw away (abandon it)

Sold it moribund/before it died Other

## Specify other.

1. **In the last 12 months, have you assisted an animal giving birth or an animal that had an abortion, retained placenta?**

Yes No

## If yes, which of these PPE do you use?

*Select all that apply)*

Face masks Gloves

Gum boots None

Overall/coat Other

## Specify other.

1. **What do you do with aborted fetus / retained placenta?**

*Select all that apply)*

Burn it Bury

Feed to dogs Leave it there

Throw in compost pit Throw in toilet

The birth was uncomplicated/normal delivery Other

## Specify other.

1. **What do you do to <b>PREVENT</b> disease/outbreak in your dairy cattle?**

*Select all that apply*

Buy new cattle from local area Change where my cattle graze Deworming

Feed supplementation

Good hygienic practices (cleanliness, disinfection, personnel hygiene, etc) Graze sick cattle separately

Keep calves separate

Keep new cattle separately

Make a shed or pen to prevent contact from other domestic and wild animals Preventive measures using modern medicine

Preventive measures using traditional medicine Regular visits by vet

Spraying

Traﬃc control

Use of milking machines to prevent mastitis Vaccination

Do nothing Other

## Specify other.

1. **When your dairy cattle <b>BECOME SICK/b>, what are the ﬁrst steps you take?**

Do nothing

Ask Neighbors/Friends for Help Consult another livestock keeper Go and buy medicine

Go/call to veterinarian or livestock health oﬃcer Go/call veterinary drug shop attendant

Isolate sick animal

My animals never get sick

Report / consult Community disease reporters Try traditional medicine

Use drugs that I have in the house Wait to see if it improves ﬁrst

Other

## Specify other.

1. **Where do you mostly/usually get your information on GENERAL DAIRY FARMING practices?**

*Top Three Choices*

I do not seek advice because I know what to do Other farmers

Government vet services Private vet services

Veterinary drug shops Veterinary laboratory Online/Internet

Training

Experience/historical exposure My boss

I never seek information on general dairy husbandry Other

## Specify other.

1. **Where do you mostly/usually get your information from when your dairy cattle get sick?**

*Top Three Choices*

I do not seek advice because I know what to do Other farmers

Government vet services Private vet services

Veterinary drug shops Veterinary laboratory Online/Internet

Experience/historical exposure

I never seek information on dairy cattle health Other

## Specify other.

1. **Do you agree with the statement that "I can easily ﬁnd a veterinarian or an animal health professional when necessary"**

agree disagree

I don't know

## How satisﬁed are you with the Government Animal Health Service Professionals?

Satisﬁed

Needs improvement Not satisﬁed

No comment

No vets in my area I never use vets

## How satisﬁed are you with the Private Animal Health Service Professionals?

Satisﬁed

Needs improvement Not satisﬁed

No comment

No vets in my area I never use vets

## What challenges do you face in trying to get animal health services from a veterinarian?

*Select All that Apply*

No challenges

Distance to veterinarian High cost

Not aware of who to contact Poor network coverage

Poor road network

Unavailable when needed (e.g., will be delayed) Other

## Specify other.

Please ask respondent "Can you please give us examples of medicines you/veterinarian used to treat your dairy cattle? (Ask to bring container if possible)"

## Select the injectables/veterinary drugs they told you or brought to you

No injectables used (Probe that both farmers and vet are not using injectables) Alamycin

Albadip 10%

albendazole tablet Berenil

Buparvaquone Butalex

I don't know

ivermectin (perimectin, hanamectin, etc) levafas diamond

levermizole Multiject norotraz Opticlox otc 10% oxyvet

Penstrep Samorin Tak Tic Terrexin veriben Other

## Specify other.

1. **Please list all the reasons you/veterinarian use injectables on your dairy cattle?**

*Select all that apply*

Boost appetite

To acclimatize to a new environment To help dairy cattle grow bigger

To help dairy cattle grow faster

To help dairy cattle increase milk production To help sick dairy cattle to get better

To prevent a single dairy cattle from getting sick

To prevent healthy dairy cattle from getting infected from sick animals I don't know

Other

## Specify other.

1. **Think about ALL your dairy cattle, how often are they administered injectable drugs in a typical month?**

One or Two 3 to 5 times

6 to 10 times

Over 10 times

Rarely, only when need arises

## About how much do you spend on injectables a month? (UGX). THIS INCLUDES ALL SERVICE FEES

*Probe those who never have to buy or use them by asking "In your experience on this farm, how much do you think your vet would ask you as all his expenses if he had to come here to inject a sick cow"*

## What season do animals mostly get sick?

Wet Dry

The cattle get sick a similar amount in wet/dry seasons

## From where do you USUALLY get your injectables from?

I've never purchased injectables a friend/peers

a shop that is not a veterinarian chemist (like a normal retail shop) a veterinarian drug shop

Drug companies

government animal health service providers open air markets

private animal health service providers Other

## Specify other.

1. **How do you USUALLY know which injectables to PURCHASE for your dairy cattle?**

*Select all that apply*

Have previously used it with success Advised by veterinarian

Advised by veterinarian chemist attendants Advised by fellow farmers

From books

Through radio or TV

Internet (Google, Facebook) Drug companies

In posters

From human pharmacy attendant Training

Other

## Specify other.

1. **Think about when you purchase injectables at veterinarian drug shops, how does it USUALLY happen?**

I know what I need and I just tell the them the medicine name

I get a prescription from qualiﬁes animal health service providers then go to purchase

I tell them symptoms of my dairy cattle and they tell me the antibiotic I need but NO INSTRUCTIONS ON USE I tell them the symptoms of my dairy cattle and they tell me the antibiotic I need and INSTRUCTIONS ON USE I never purchase injectables from a veterinary shop

## When you buy injectables for your dairy cattle from a veterinary drug shop, does the seller USUALLY require you to have a prescription?

never/rarely sometimes almost always

I never purchase injectables from a veterinary shop

## When using veterinary drugs on dairy cattle, what is the MOST IMPORTANT sources of instructions on use (kind, dose, length of treatment) READ CHOICES

*Select top 3*

A trained government animal health professional A trained private animal health professional

Fellow farmers Read the label

Use my own judgement and experience Veterinary drug shops

I never use veterinary medicines

# Treatment

1. **When your dairy cattle need injectables, who administers the drug?**

**Owner**

**Fellow farmer/friends Other household members Worker**

**Gov Veterinarian Private Veterinarian**

No Yes NA, no injectables used

## If the ﬁrst treatment does not lead to any improvement in the dairy cattle, what do you/veterinarian do?

*Select all that apply*

Ask God/higher power for help

Called a vet (for ﬁrst time if self-administered) Called back the same vet

Called for advice from another farmer Called for advice from another vet Do nothing

Emergency slaughter I increased the dose I sell the animal

I stopped using the drug

Prolong the treatment period using the same drug Supplement with traditional medicine

Tried a diﬀerent injectable

No treatment failure encountered Animal died during the ﬁrst treatment

## In the last year, did any dairy cattle die that were getting treated with injectables?

Yes No

## If yes, why do you think it did not heal after treatment?

The drugs were not strong/counterfeit/poor dosage The disease was too strong

Wrong diagnosis/treatment Other

## Specify other.

1. **Are there times when a dairy cattle needs a LARGER DOSE of an injectable than the normal dose?**

yes no

Don't Know

## Brieﬂy record why they/veterinarian DO give a larger dose

1. **Are there times when dairy cattle need a SMALLER DOSE of an injectable than the normal dose?**

yes no

Don't Know

## Brieﬂy record why they/veterinarian DO give a smaller dose

1. **Have there been situations where you needed to use expired veterinary drugs/injectables?**

yes no

Don't Know

## Please describe the situation where you/veterinarian had to use expired drugs

1. **If the injectables you have are expired, what do you USUALLY do with them?**

Give to neighbors and friends I do not check the expiry date

I do not worry about the expiry date

I rinse out chemical and then throw away Return them back to where you bought them Throw away in compost pit

Throw away in latrine

Will not use them and bury

Will use them until they are ﬁnished

I don not personally use or stock injectables / never had expired injectables Other

## Specify other.

1. **Do you agree with the statement that "it is important to get consultation from an animal health professional like a veterinarian before giving injectables to the animals?**

agree disagree

I don't know

## Do you agree with the statement that "You can stop giving a dairy cow a full course of injectables if their symptoms are improving."

*Refer to the vet`s practice. E.g say "Ïf the treatment is for 5 days, and the animal heals in 3 days, and the vet says it is not necessary to continue injecting".*

agree disagree

I don't know

## Do you agree with the statement that "if injectables are given too often then they might stop working?"

agree disagree

I don't know

## Do you agree with the statement that "giving dairy cattle that are not sick injectables will PREVENT them from becoming sick in the future"

*Give a scenario to clearly depict prophylactic use of drugs E.g "This is April and your cows are healthy now. However, last year in May, it was dry and you had an outbreak of East coast fever. It is now starting to get hot, so you anticipate they may fall sick around the same time this year. Do you think injecting them now would prevent them from getting fever in June?*

agree disagree

I don't know

## Do you agree with the statement that "giving dairy cattle injectables can help them grow BIGGER and FASTER"

agree disagree

I don't know

Now, I'd like to ask you a few questions about tick borne diseases

## Are tick borne diseases getting more diﬃcult to treat?

yes no

Don't Know

## What tick diseases are getting harder to treat?

I don't know the disease, just fever Anaplasmosis

Babesiosis

East Coast Fever (Makebe) Heartwater

Other

## Specify other.

1. **How do you PREVENT ticks and tick diseases from impacting your dairy cattle?**

*Select all that apply*

Applying grease Dipping

Keeping animal separate or at home to prevent mixing Make sure pen is clean/sanitary

Spraying Other

## Specify other.

1. **If spraying does not kill ticks, what do you do?**

alternate acaracides

Increase concentration of acaracides mixing acaracides with pesticides No resistance encountered

Spraying always kills ticks I never spray for ticks Other

## Specify other.

1. **If the cattle is not healing from the DISEASE, what do you do?**

I don't do anything I call the vet (probe by saying "if vet does not come what do you do?" Change to other drugs

Increase dosage Sell oﬀ

Switch to traditional medicine Other

## Specify other.

1. **Why do you believe tick DISEASES are getting more diﬃcult to treat?**

drugs are poor

the diseases are getting stronger I don't know

Other

**Specify other.**

# Now, I'd like to ask some questions about vaccines

## During the last 3 years, what diseases have your cattle been vaccinated for?

*Select all that apply*

Anthrax Black Leg FMD

LSD CBPP RVF

My cattle are not vaccinated against any disease I don't know what vaccines are

## Where do you USUALLY source your vaccines from?

*Select all that apply*

Private Veteriniarian

Government Veterinarian

vaccination campaign/community outreach Dairy cooperatives

I don't buy or give vaccines to my animal Other

## Specify other.

1. **Who USUALLY vaccinates the dairy cattle?**

Govt. animal health service provider Myself/fellow farmers

private animal health service providers Veterinarian Chemist attendant

## Do you know what vaccines do?

they prevent disease for the lifetime of the animal they prevent disease for sometime

they treat disease

## What challenges do you normally face with getting vaccinations for dairy cattle?

*Select all that apply*

No challenges

High cost of administering vaccination High cost of vaccine

Inadequate personnel available to give vaccine Lack of cold chain

lack of information on what vaccines and when to use Not applicable, do not give vaccines

Unavailability in local area Other

## Specify other.

1. **Have you ever experienced the following soon after your cow received a vaccination (about 3 days)?**

*List all choices*

Sickness Death Abortion Other

## Specify other.

1. **Do you agree with that statement that "using vaccines can REDUCE the use of veterinary drugs?"**

agree disagree

I don't know

# Now, I'd like to ask some questions on your milking practices

## What preparation do you do before milking?

*Remember, DO NOT LIST just WAIT FOR FARMER to tell you*

**Wash your hands with soap Washing of milking container Cleaning the udder with clean water**

**Dry the udder with a dry clean towel / cloth**

**Milk the healthy teat ﬁrst (if mastitis) Milk the healthy cows ﬁrst**

**Separate milking area Application of milking salve Get a clean towel**

1. **Do you use a sieve before or after milking?**

Yes No

No Yes Does not apply

## How do you USUALLY clean equipment before milking?

*Select all that apply*

Boiled water Cold water Disinfectant I do not clean Soap

Sun Dry Other

## Specify other.

1. **How often do you clean the milking equipment?**

Never (I do not clean the milking containers) Less than once per day

Only once per day Yes, before every use

## Do you clean milking containers between cows?

No

No, only one cow Yes

## Has your milk ever been rejected by collectors, consumers etc?

*Select all that apply*

No

Yes, because of bad odor Yes, discolouration

yes, history of quick pillage Yes, milk was watered down

Yes, presence of physical material Yes, price

Other

## Specify other.

1. **In what season do you experience the most rejections?**

Dry Rainy

No diﬀerence in rejections across seasons No rejections

## What did you do with the rejected milk?

*Select all that apply*

Hand out to the needy self-consume Look for other milk collectors

Sell to household consumers at reduced price as fresh as milk Sell to household consumers at regular price as fresh milk Sell to shops/restaurants

Throw away (dispose as waste) Value addition (yoghurt, ghee)

## Do you agree with the statement that "Consumption of raw milk can aﬀect human health negatively"

agree disagree

I don't know

## If they sell meat from ANY ANIMAL, ask "has your meat ever been rejected?"

No Yes

Not applicable, never sold meat

## In what season do you experience the most meat rejections?

Dry Rainy

No diﬀerence in rejections across seasons No rejections

Now, I'd like to ask you some questions about antibiotic or antimicrobial resistance

## Have you ever heard of antibiotic or antimicrobial resistance?

Yes No

## What did you hear about antibiotic/antimicrobial resistance?

1. **Who told you about antibiotic/antimicrobial resistance?**

Agrovets

A trained vet or livestock health oﬃcer Learned in school

Family

Friend or Neighbor

From media (social media, print, radio and TV, Church or Mosque ) Read the label

From veterinary books

I never use veterinary medicines

## Has an animal health worker/veterinarian ever told you about the risks of using veterinary drugs incorrectly (wrong dosage/treatment period, wrong dug ﬁr disease, too often, etc)?

Yes No

## Do you know what antibiotic/antimicrobial residues are?

*Please break it down into local terms*

Yes No

## Do you agree with the statement that "after using veterinary drug on an animal, you should wait to use milk from it"

agree disagree

I don't know

## What do you USUALLY do with milk from animal products under current treatment with injectables or during withdrawal period?

Give to calf

Give to other animals Sell it

Throw it away

Use for home consumption Not applicable

Other

## Specify other.

1. **Has a veterinarian ever told you about withdrawal periods: that is, you have to wait to sell or eat a meat/egg/milk after giving animal medicine?**

Yes No

## Can animals transmit disease to humans?

yes no

Don't Know

# Now, I'll ask you a few questions about farm management

## Does anyone in the household have any training/workshop related to animal health?

Yes No

## What did the trainings cover? Please brieﬂy describe

1. **Do you take farm records? If yes, please list what types.**

*Select all that apply*

I don't keep any records Breeding Records

Feed Records

General Financial Records

I used to keep records but stopped Medicine used

Mortality

Production Records (milk, eggs, calves, etc) Other

## Specify other.

**Was the veterinarian sitting with you during interview/do you think he/she could hear responses?**

Yes No

Tell the informant that you appreciate their time and that their responses will help us succeed in our project. And tell them after we analysed the data we will invite them to a workshop were we share the data.

Survey End.
