# Supplementary material for: Minding the knowledge-action gap: Results from a mixed-methods study of antimicrobial use among dairy farmers in central Uganda
Source: PLoS One. 2026 Jan 9;21(1):e0339969. doi: 10.1371/journal.pone.0339969 (PMC12788652; doi:10.1371/journal.pone.0339969)
Supplement: Attachment — Submitted filename: Minding the Knowledge review.pdf [file pone.0339969.s006.pdf]

Minding the Knowledge-Action Gap: Results from a Mixed-Methods Study of Antimicrobial Use Among Dairy Farmers in Central Uganda

This mixed-methods study examines knowledge, attitudes, and practices related to antimicrobial use among Ugandan dairy farmers using surveys and focus groups. While addressing an important research gap in antimicrobial resistance in low-income agricultural settings, the manuscript has some methodological and analytical limitations that require revision before publication consideration.

Throughout: Inconsistent terminology and causal language where correlational terms would be appropriate. Key findings are buried within methodological details rather than clearly highlighted.

Tables: Generally clear but lack important methodological details such as reliability coefficients for constructed scales and detailed missing data patterns.

Missing Information: No power analysis, sample size justification, inter-rater reliability for qualitative coding, or comprehensive missing data analysis provided.

Abstract and Introduction

"We found that (1) farmers' AMU practices are shaped by attitudes toward veterinarians..." - The cross-sectional design cannot establish causality, yet the language implies causal relationships throughout. This should be "farmers' AMU practices were associated with attitudes toward veterinarians..."

"knowledge about AMR did not predict better practices" - This overstates findings from exploratory regression models that explain limited variance (R^2 = 0.18-0.20 for model 2 and 1 respectively).

Line 19: The definition of metaphylaxis is incomplete and potentially misleading. They define it as "clinically healthy animals sharing premises with symptomatic animals," which is more of a description of the situation rather than the actual antimicrobial intervention. The accepted veterinary definition of metaphylaxis is the treatment of clinically healthy animals that have been exposed to infectious agents, typically when some animals in the group are already showing clinical signs of disease. It's a targeted group treatment strategy.

Lines 25-26, 28-29, 32-33: The repeated use of "LICs and LMICs" throughout the manuscript reflects a misunderstanding of standard World Bank classification terminology. "LMICs" (low- and middle-income countries) already includes low-income countries as a collective term for all non-high-income countries. The redundant phrasing "LICs and LMICs" should be replaced with simply "LMICs" or "low- and middle-income countries (LMICs)" on first usage.

Lines 25-36: The comparison between Uganda and high-income countries lacks nuance about different production systems and contexts that might explain these differences beyond just veterinarian ratios.

Lines 55-58: The claim about "absence of any prior KAP study" in Uganda's livestock sector appears overstated without comprehensive literature search evidence. Consider qualifying this statement. For example, here is a quick search result; Kahunde M. A.Odoch T.Owiny D. O.Kankya C.Kaelin M. B.Hartnack S. (2023). Knowledge, attitudes and practices towards antibiotic use and resistance in Kyegegwa district, Uganda – a questionnaire study. medRxiv. 10.1101/2023.04.06.23288253; https://www.researchgate.net/publication/389052753_Smallholder_farmers'_knowledge_attitude_and_practices_towards_use_and_disposal_of_veterinary_antibiotics_in_Kassanda_District_Uganda_-_A_cross_sectional_survey

Line 72: "failure, and even the emergence of "Multidrug-resistant bacteria" that render no drugs" - This phrase is awkward and unclear. Should be rephrased for clarity.

Methodology

Lines 83-89: The convenience sampling approach through veterinary extension workers introduces systematic selection bias not adequately addressed. Farmers connected to extension services may differ in knowledge, practices, and veterinary access from the broader population.

Lines 90-95: The definition of "smallholder" as 1-15 cattle appears arbitrary without supporting rationale from literature or local context. What constitutes "smallholder" varies significantly across regions and should be justified.

Lines 111-112: Only three focus group discussions are insufficient for understanding complex behavioural phenomena. No discussion of data saturation is provided, and this limitation significantly undermines the qualitative component's validity.

Lines 119-120: Survey duration of 45-90 minutes suggests potential respondent fatigue that could affect data quality, yet this substantial variation is not addressed or explained.

Lines 135-161: Multiple statistical methodology concerns: Stepwise regression with p=0.1 cutoff increases Type I error risk and is problematic for exploratory analysis. The creation of composite scales without psychometric validation (reliability, construct validity) is concerning. The "antimicrobial misuse scale" combines conceptually different behaviours without theoretical justification for equal weighting.

Lines 157-158: "Regression diagnostics were run for all models, generally indicating good model fit (see Annex A)" - No Annex A is provided. Either include diagnostic results or remove this reference.

Lines 158-159: Listwise deletion of missing data without analysis of missingness patterns is problematic, especially given the exploratory nature and potential systematic differences in missing responses.

Table 1: Variable definitions lack sufficient operational detail for replication. For example, how exactly was the "antimicrobial misuse scale" calculated and validated?

Results

Lines 193-201: Demographic distribution (60% male, high education levels) suggests potential sampling bias, but no comparison with broader population characteristics is provided to assess representativeness.

Lines 207-210: Several percentage calculations in Table 2 are incorrect. For example, 'Owner treats: 25.8%, 98 obs' should be 23.5% (98÷417), 'Prescription obtained never/rarely: 57.5%, 217 obs' should be 52.0% (217÷417), and similar errors exist for peer and worker treatment percentages. These calculation errors require correction throughout the table.

Lines 234-245: Statistical interpretation problems. "Age was negatively related to misuse" implies causation from correlation. The coefficient of -0.13 may lack practical significance despite statistical significance.

Lines 247-250: The models explain limited variance (R^2 = 0.18-0.20), indicating that most factors influencing antimicrobial use remain unidentified. This limitation undermines claims about identifying key correlates.

Lines 267-310: While qualitative insights are valuable, the limited sample (3 FGDs) undermines claims about thematic saturation. Quotes lack sufficient context about speaker characteristics and farm contexts.

Discussion

Lines 325-366: The authors make policy recommendations about private veterinarians without acknowledging how their methodological limitations (convenience sampling, cross-sectional design) affect the generalisability of these recommendations. The Rwanda comparison lacks critical analysis of contextual differences.

Lines 417-463: The future research directions don't address their own study's methodological shortcomings. No mention of needs for probability sampling, longitudinal designs, scale validation, etc. They focus on general research directions rather than building on lessons from their study's limitations.

Lines 464-488: The limitations section significantly understates problems. While they mention cross-sectional design and convenience sampling, they completely omit:

- Measurement validity of composite scales (not mentioned at all)
- Detailed discussion of selection bias implications
- Generalisability constraints (only briefly mentioned)
- More thorough discussion of social desirability bias impacts

Lines 490-499: The conclusion uses causal language ("influence suboptimal antimicrobial use") when only correlational relationships were established, and makes broad policy recommendations despite methodological limitations.
