# Supplementary material for: Minding the knowledge-action gap: Results from a mixed-methods study of antimicrobial use among dairy farmers in central Uganda
Source: PLoS One. 2026 Jan 9;21(1):e0339969. doi: 10.1371/journal.pone.0339969 (PMC12788652; doi:10.1371/journal.pone.0339969)
Supplement: Attachment — Submitted filename: Reviewer comments.docx [file pone.0339969.s007.docx]

**REVIEWER COMMENTS**

Introduction.

The reader might find it difficult to understand the statement in lines 3-5. Provide more context either by citing sources or directly linking it to the main objective.

Even though it may be implied in line 44, it is not clear where Uganda falls (economic classification)

Check citation in line 49

Line 59. The sentence seems to be missing a connecting word or needs rephrasing entirely

Methodology.

Consider stating, clearly, the study design in the methodology

As the section flows, it should indicate specific data collection tools for each component of the ‘mixed methods’ approach. March 15th to April 17th may also need to be moved to an appropriate section, as it speaks to the retrospective nature of the study.

Given the nature of the sampling technique, was there any need for sample size estimation? If yes how was the required sample size estimated?

The sampling unit (either a household or individual farmers operating small or medium-sized dairy cattle farms) needs to be clearly and early stated

This section could benefit from more detail and a clearer flow. More specifically, there’s no clear transition between the qualitative and quantitative sections in terms of:

- Which part of the study used this recruitment…. “Recruitment involved local veterinarians and peer referrals”
- Data collection tools and pre-data collection preparations.
- The interchangeable use of the term “survey” for both questionnaires (quantitative) and FGDs (qualitative) may confuse the reader. Separate these and allow each to flow independently. Elaborate further on the sampling strategy. The fact that these were researcher/enumerator-administered questionnaires needs to come out earlier in the methodology

Put the sampling method in the appropriate section

Were FGDs conducted before questionnaires were complete? Could this, in any way, have impacted the structure of the questionnaire or introduced any bias?

What tool/software was used to analyze the quantitative data

Add a concise stepwise approach to achieving the first objective in the data analysis section

Kindly build up on how you got to the models in the data analysis process

It would be prudent to have a few mentions of some major statistical tests useful in assessing, e.g, correlations……

Results

The results are well expressed. Results from the models are excellent

Was there any internal validity or reliability approach used?

How was the model’s goodness of fit assessed? How was the model’s general performance?

Discussion

Addresses the objectives well
